# Isolation, Identification, and Optimization of Culture Conditions of a Bioflocculant-Producing Bacterium* Bacillus megaterium* SP1 and Its Application in Aquaculture Wastewater Treatment

**DOI:** 10.1155/2016/2758168

**Published:** 2016-10-20

**Authors:** Liang Luo, Zhigang Zhao, Xiaoli Huang, Xue Du, Chang'an Wang, Jinnan Li, Liansheng Wang, Qiyou Xu

**Affiliations:** Heilongjiang River Fisheries Research Institute, Chinese Academy of Fishery Sciences, Harbin 150070, China

## Abstract

A bioflocculant-producing bacterium,* Bacillus megaterium* SP1, was isolated from biofloc in pond water and identified by using both 16S rDNA sequencing analysis and a Biolog GEN III MicroStation System. The optimal carbon and nitrogen sources for* Bacillus megaterium* SP1 were 20 g L^−1^ of glucose and 0.5 g L^−1^ of beef extract at 30°C and pH 7. The bioflocculant produced by strain SP1 under optimal culture conditions was applied into aquaculture wastewater treatment. The removal rates of chemical oxygen demand (COD), total ammonia nitrogen (TAN), and suspended solids (SS) in aquaculture wastewater reached 64, 63.61, and 83.8%, respectively. The volume of biofloc (FV) increased from 4.93 to 25.97 mL L^−1^. The addition of* Bacillus megaterium* SP1 in aquaculture wastewater could effectively improve aquaculture water quality, promote the formation of biofloc, and then form an efficient and healthy aquaculture model based on biofloc technology.

## 1. Introduction

Bioflocculant is an active substance produced by growing microorganisms and is composed of macromolecular polymers, such as glycoprotein, polysaccharide, protein, cellulose, and nucleic acid [1–3]. Bioflocculant offers many advantages for suspended solids (SS) removal, such as high security and efficiency, low cost, being nontoxic, and producing no secondary pollution for the environment [4–10]. The use of bioflocculant for SS removal has been widely used in industrial, domestic, and building material and livestock wastewater treatment as a new water treatment agent [11–14]. Although there were some general reports of bioflocculant in wastewater treatment, relevant research and application of bioflocculant in aquaculture wastewater treatment have rarely been reported.

In recent years, the aquaculture industry had developed rapidly with a worldwide presence, especially in China. However, low feeding utilization rates caused approximately 75% of the aquaculture feed to remain as nitrogen and phosphorous in the wastewater [15]. Aquaculture wastewater was discharged arbitrarily into rivers, lakes, and ocean, resulting in eutrophication and even red tide disasters. Many efforts have sought to reduce and regulate the generation and emission of aquaculture wastewater, such as upscaling aquaculture wastewater treatment by microalgal bacterial flocs [16], application of probiotics in carp aquacultures [17], removal of organic matter from polluted coastal waters by floating bed phytoremediation [18], and the application of artificial wetlands in multistage aquaculture wastewater purification [19]. Biofloc technology as one of the most advanced aquaculture technology models has been widely applied in shrimp, tilapia, and carp pond cultures. Using biofloc technology produced more aquaculture products without significantly increasing the usage of the basic natural resources of water and land, minimized damage to the environment, and provided an equitable cost/benefit ratio to support economic and social sustainability [20–24].

Compared with industrial and domestic wastewater, aquaculture wastewater is mainly composed of carbon, nitrogen, phosphorous, and other nutrients. Aquaculture wastewater also has its own characteristics, such as fewer poisonous metal materials and lower concentrations of nitrogen, phosphorous, SS, and chemical oxygen demand (COD). Therefore, bioflocculant-producing bacteria could feasibly be added to ponds and then used to effectively treat aquaculture wastewater. The aim of the present study was basically developed by two sections. The first section was to isolate and identify a bioflocculant-producing bacterium from fish pond and to optimize its culture conditions. In the second section, the bioflocculant produced by* Bacillus megaterium* SP1 was applied in the aquaculture wastewater treatment to reduce the COD and inorganic nitrogen, promote the formation of biofloc, improve the utilization rate of nitrogen, and ultimately form a highly efficient and healthy aquaculture model suitable for China's pond aquaculture.

## 2. Materials and Methods

### 2.1. Biofloc Samples and Isolation of Bioflocculant-Producing Bacterium

Biofloc samples were collected by Imhoff cones from the carp biofloc technology pond at Hulan experimental station of Heilongjiang River Fisheries Research Institute in Heilongjiang Province, China (45.97°N, 126.63°E). The samples were stored at 4°C in sterile containers. First, each biofloc sample was homogenized and serially diluted in sterile water. Second, each dilution was spread on enrichment medium and incubated at 30°C for 72 h. Strains with different colony morphology were taken and repeatedly cultivated for purification, and the single pure colony was saved for later use. Third, each pure colony was spread on fermentation medium and cultured at 30°C in a rotary shaker at 160 r min^−1^ for 72 h. The culture broth was used to determine for flocculating efficiency. The strain with the highest flocculating efficiency and good several subcultures was selected as the bioflocculant-producing bacterium for further study. Kaolin suspensions at a concentration of 5 g L^−1^ were then used to evaluate the flocculating capability of a series of the culture. Among them, the enrichment medium included beef extract (3 g L^−1^), peptone (10 g L^−1^), and NaCl (5 g L^−1^) and was amended with 1.8% agar. The fermentation medium included glucose (20 g L^−1^), KH_2_PO_4_ (2 g L^−1^), K_2_HPO_4_ (5 g L^−1^), MgSO_4_·7H_2_O (0.5 g L^−1^), (NH_4_)_2_SO_4_ (0.2 g L^−1^), NaCl (0.1 g L^−1^), urea (0.5 g L^−1^), and yeast extract (0.5 g L^−1^).

### 2.2. PCR Amplification and Phylogenetic Analysis

The bacterial genomic DNA of strain SP1 was extracted using the E.Z.N.A.® Bacterial DNA Kit (Omega Bio-Tek, Inc., USA). PCR amplification of the 16S rDNA was performed with universal primers (27F, 5′AGAGTTTGATCCTGGCTCAG3′, and 1492R, 5′GGTTACCTTGTTACGACTT3′). The amplification system was composed of a total volume of 50 *μ*L containing 3 *μ*L of total DNA, 1 *μ*L of 27F, 1 *μ*L of 1492R, 1 *μ*L of dNTP, 5 *μ*L of 10x buffer, 0.6 *μ*L of Taq DNA polymerase, and 38.4 *μ*L of ddH_2_O [25]. The reaction conditions were as follows: 94°C for 4 min followed by 30 cycles of denaturation at 94°C for 1.5 min, annealing at 55°C for 1 min, and primer extension at 72°C for 1.5 min and a final extension at 72°C for 10 min [26]. PCR products were purified using PCR production purification kit, and the purified PCR products were sent to Suzhou GENEWIZ Biotechnologies Co. Ltd. (China) for sequencing. The sequence results were submitted (accession number: KU529280) to the GenBank database. Software MEGA 6.0 was used to construct a phylogenetic tree by the neighbor-joining method [27].

### 2.3. Identification with Biolog GEN III MicroStation System

The Biolog GEN III MicroStation System is an automated microbial identification system based on aerobic metabolic activities without the labor-intensive requirements of conventional strips or panels. The strain SP1 was characterized using Biolog GEN III microplate (Biolog Inc., Hayward, CA, USA). GEN III plate contains 95 different carbon substrates, which is based on interpreting patterns of sole-carbon substrate utilization indicated by color development in a 96-well microtiter plate. By analyzing the similarity of the metabolic fingerprints between SP1 and standard strains in the kinetic database by Biolog Retrospect 2.0 Data Management Software, the strain was identified. Among them, when SIM is > 0.5 and DIST is < 5.00, this is a more satisfactory result [28].

### 2.4. Analysis of Flocculating Efficiency

The flocculating efficiency of the bioflocculant produced by the bacterial culture was measured by kaolin suspension. In general, 2 mL of the bioflocculant (the culture broth of strain SP1), 5 mL of CaCl_2_ (1%, w/v), and 93 mL of kaolin suspension were mixed in a 200-mL beaker. The mixture was stirred at 180 r min^−1^ for 1.5 min and at 80 r min^−1^ for 3 min with a vortex mixer (QL-861, Shanghai Jingmi Instrument Co., Ltd., China) and then kept still for 10 min. The supernatant portion was absorbed to determine its optical density (OD) at 550 nm by a 752 spectrophotometer [4]. The steps for the blank control were similar to the above steps except that the culture broth of strain SP1 was replaced with distilled water. All assays were conducted in three duplicates. The flocculating efficiency was defined and calculated as follows:

(1) $$\begin{array}{c} \text{Flocculating efficiency}\left(\;\%\;\right)=\frac{\left(A_{0}-A\right)}{A_{0}}\times 100, \end{array}$$

where *A*_0_ and *A* were OD_550_ of the blank control and of the supernatant, respectively.

### 2.5. Optimization of Culture Conditions

Experiments were designed in which carbon and nitrogen sources of fermentation medium were replaced by various carbon and nitrogen sources in fresh fermentation medium. The one-way medium was used to determine the flocculating efficiency for kaolin suspension and to select the optimum carbon and nitrogen source for strain SP1.

According to the one-way experiment results, specific carbon and nitrogen sources in the original fermentation medium were replaced by the optimum carbon and nitrogen sources for optimal performance. The four factors of carbon source, nitrogen source, initial pH, and temperature were the major factors influencing flocculation and were selected to design L_16_(4^5^) orthogonal experiment. The optimum culture conditions were obtained by the analysis of the orthogonal experiment results.

### 2.6. Preliminary Application in Wastewater Treatment of Aquaculture

The aquaria (90 × 55 × 45 cm) selected for the experimental tanks had 100 L of three types of water (aquaculture wastewater, Hulan river water, and urban domestic wastewater) with continuous aeration. Culture broth of strain SP1 containing 1 × 10^7^ CFU mL^−1^ was added to the aquaria water samples by an adding ratio of 1 × 10^4^ CFU mL^−1^ to evaluate its effect on wastewater, especially by comparing how the data changed before and after inoculation. The experiment was divided into three groups, each group containing three duplicates. The indexes of chemical oxygen demand (COD), total ammonia nitrogen (TAN), suspended solids (SS), and volume of biofloc (FV) were determined.

### 2.7. Analytical Methods

The COD and SS were determined using the methods given by the National Standard of China, TAN was determined by the YSI Professional Plus (YSI Incorporated, Yellow Springs, USA), FV was determined by sampling 1000 mL pond water into a series of Imhoff cones [29], and the volume of the floc plug accumulating on the bottom of the cone was determined 15 min following sampling [20].

### 2.8. Statistical Analysis

Data analysis was performed by one-way ANOVA using SPSS 17.0 software for Windows. Duncan's multiple range tests were used to identify differences among experimental groups, and the level of statistical significance was accepted as *P* < 0.05.

## 3. Results and Discussion

### 3.1. Isolation of Bioflocculant-Producing Bacterium

Approximately 48 isolates were selected from the biofloc samples (Table 1). However, only six strains with flocculating efficiency exceeding 80% were able to actively flocculate kaolin suspension, as measured after five or more subcultures. Among them, the bacterium named SP1 with the highest flocculating efficiency was selected as the bioflocculant-producing bacterium for further study.

### 3.2. Identification and Characterization of Bioflocculant-Producing Bacterium

**Figure 1:**
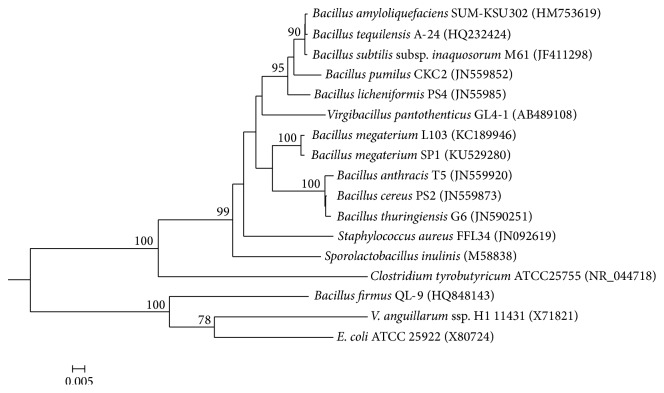
Neighbor-joining tree between* Bacillus megaterium* SP1 and its phylogenetically closest microorganisms based on the 16S rDNA. The scale bar indicates 0.005 substitutions per nucleotide position.

Strain SP1 was a circular, smooth, white, rod-shaped, Gram-positive bacterium with fermented liquid that was brown and turbid. Molecular analysis based on 16S rDNA confirmed the strain SP1 to be a* Bacillus* sp.; therefore, it was named* Bacillus* sp. SP1. The nucleotide sequence obtained in the present study had been submitted to GenBank and assigned accession number KU529280. In the phylogenetic tree, strain SP1 and the other closest* Bacillus* strains were grouped together (Figure 1). Strain SP1 was further identified using the Biolog GEN III MicroStation System, which was Biolog's latest generation product for the testing and microbial identification of aerobic Gram-negative and Gram-positive bacteria because they were in the same test panel; Gram stain and other pretests were no longer needed [30]. The results showed that strain SP1 was* Bacillus megaterium* (probability 59.6%, SIM 0.596, and DIST 5.883) based on the carbon source metabolic characteristics (Table 2). Therefore, this strain was named* Bacillus megaterium* SP1.

Bioflocculants were produced by many microorganisms widely distributed in soils and waters [31]. More than 70 bioflocculant-producing microorganisms have been reported, such as* Bacillus subtilis* [5],* Bacillus firmus* [32],* Bacillus licheniformis *[33],* Bacillus mucilaginosus* [2],* Proteus mirabilis* [34], and* Klebsiella *sp. [35]. However, bioflocculant produced by* Bacillus megaterium* and its application in wastewater treatment have rarely been reported. It was found that the extracellular polymeric substances (EPS) from* Bacillus megaterium* TF10 exhibit a high flocculation activity [36]. One report of a* Bacillus megaterium* strain producing a biodegradable flocculant was observed for turbidity and arsenic removal during growth [37]. Another bioflocculant produced by* Bacillus megaterium* YWO-5 was used for wastewater treatment [38]. In this work, the highly efficient bioflocculant-producing bacterium* Bacillus megaterium* SP1 was especially isolated from biofloc samples of aquaculture ponds for the purpose of accelerating biofloc formation and improving the water quality in aquaculture ponds.

### 3.3. Optimization of Culture Conditions

#### 3.3.1. The Selection of the Optimum Carbon Source

**Figure 2:**
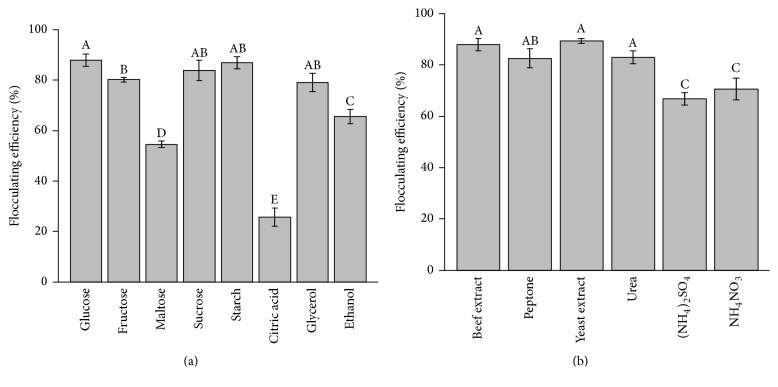
Effects of various carbon sources (a) and nitrogen sources (b) on flocculating efficiency. The bars are the respective standard deviations (*n* = 3), and values in the line with different superscript letters are significantly different (*P* < 0.05).

Carbon source is a carbonaceous material used in microbial cells to supply energy for microbial growth, reproduction, and movement. To investigate the effect of various carbon sources on flocculating rate (in a kaolin suspension) under optimal culture conditions, the carbon source glucose (carbon content 0.4%) was used as fermentation medium in the control group and was replaced by various carbon sources (sucrose, fructose, maltose, soluble starch, citric acid, glycerol, and ethanol at the same concentration; other components remain unchanged) (Figure 2(a)). It was evident that glucose, fructose, sucrose, and soluble starch were suitable for bioflocculant production with the flocculating efficiency exceeding 80% after 72 h cultivation. The strain SP1 adapted well to a variety of carbon sources; the specific flocculating rates of glucose and soluble starch were 87.9% and 86.8%, respectively. Therefore, glucose was chosen as the optimum carbon source of strain SP1 because it had the highest flocculating activity and it has the lowest cost.

#### 3.3.2. The Selection of the Optimum Nitrogen Source

Nitrogen sources provide the raw material for microbial amino acid synthesis. The effect of various nitrogen sources on the flocculating efficiency (in a kaolin suspension) after 72 h cultivation was observed. Beef extract, peptone, urea, (NH_4_)_2_SO_4_, and NH_4_NO_3_ replaced yeast extract (nitrogen content 0.03%) at the same concentration which was shown in Figure 2(b). The flocculating efficiency of six different nitrogen sources ranged from 66.88% to 89.37% and illustrated that certain nitrogen sources had a greater influence on the flocculating activity for the strain SP1. Specifically, beef extract and yeast extract produced bioflocculant with the flocculating efficiency exceeding 85% after 72 h cultivation. As a result, the beef extract was chosen as the best nitrogen source of strain SP1 for further study because of its high flocculating efficiency, complicated composition, and abundant nutrition.

### 3.4. Optimization of Culture Medium and Culture Conditions by Using Orthogonal Experiments

Orthogonal test factors and levels for flocculation of strains SP1, including glucose, beef extract, culture temperature, and culture medium initial pH values (with A, B, C, and D), were shown in Table 2. Orthogonal experiments were conducted to determine the optimal culture conditions. Orthogonal experimental results were shown in Table 3. The results of the range analysis suggested that the flocculating efficiency was influenced by the following factors in the descending order: glucose > beef extract > culture temperature > culture medium initial pH.

Microbial growth is influenced by culture medium composition and various survival factors. Lower concentration of carbon and nitrogen sources keeps strains such as SP1 from getting enough nutrients, thus affecting its growth and flocculating efficiency of the bioflocculant. In contrast, higher concentrations of carbon and nitrogen sources can make higher concentrations of inhibitory substances that negatively affect microbial growth as well as the flocculating rate of bioflocculant [31, 39, 40]. Microbial activity and metabolism are related to temperature; the suitable temperature is beneficial to microbial growth and metabolic rate. It was generally believed that the optimum temperature for bioflocculant formation was between 25 and 35°C, with low temperatures slowing bacterial growth and high temperatures changing the structure of the protein or peptide chain included in the bioflocculant (leading to degeneration) [41]. Initial pH also can affect the growth of bioflocculant-producing bacteria; in general, the optimal pH value of bioflocculant-producing bacteria is from neutral to weak alkaline. For different microorganism, the optimum pH value is not the same [42].

In this study, the optimal factor combination for flocculating efficiency from the result above was A_3_B_2_C_3_D_1_: 20 g of glucose, 0.5 g of beef extract, culture temperature of 30°C, and a medium initial pH of 7. Under these optimum culture conditions, the flocculating efficiency of bioflocculant produced by strain SP1 for kaolin suspension was 94.32%.

### 3.5. Preliminary Application in Wastewater Treatment of Aquaculture

**Figure 3:**
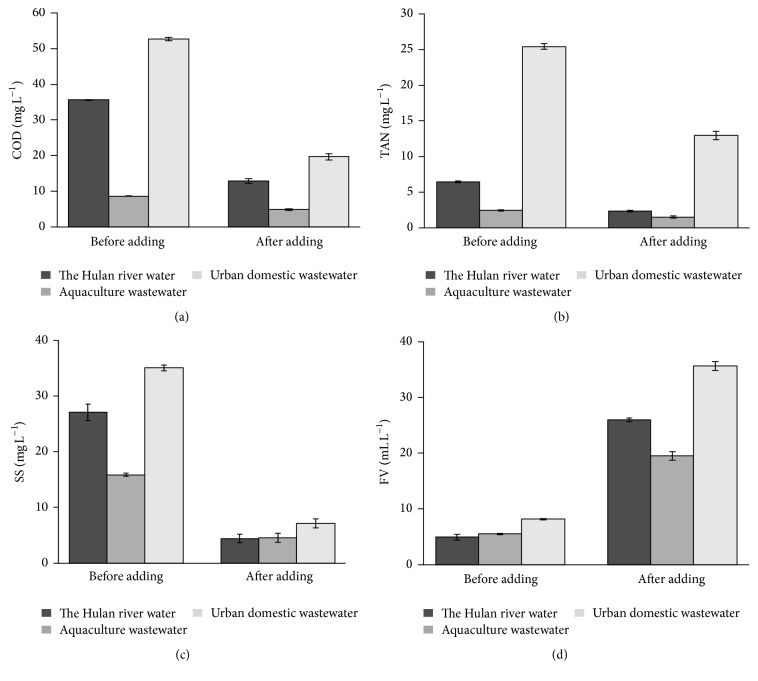
Effects of culture broth of strain SP1 on the chemical oxygen demand (COD), total ammonia nitrogen (TAN), suspended solids (SS), and volume of biofloc (FV) of aquaculture wastewater, the Hulan river water, and urban domestic wastewater. The bars are the respective standard deviations (*n* = 3).

Based on the orthogonal experiment results, two types of wastewater and Hulan river water were treated under optimal conditions (A_3_B_2_C_3_D_1_), and the results were shown in Figure 3. The aquaculture wastewater quality after the treatment improved significantly. COD decreased from 35.6 to 12.8 mg L^−1^ (*P* < 0.05), TAN decreased from 6.43 to 2.34 mg L^−1^ (*P* < 0.05), SS decreased from 27.1 to 4.43 mg L^−1^ (*P* < 0.05), and FV increased from 4.93 to 25.97 mL L^−1^ (*P* < 0.05). Under optimal culture conditions, the strain SP1 produced bioflocculant for aquaculture wastewater with a better purification effect: the removal rate of COD was from 44.19% to 64.04%, the removal rate of TAN was from 33.83% to 63.61%, and the removal rates of the SS were all over 70%. Interestingly, the FV ratio increased from 255.25% to 426.35%, which demonstrated that adding the culture broth of strain SP1 to wastewater could effectively accelerate the formation of biofloc. Adding SP1 could not only solve the problem of accumulation of harmful substances in aquaculture water but also promote the volume of biofloc which could be eaten by fish, improve the efficiency of protein generation in fish, reduce the feed demand for fish, and increase the income gained from aquaculture [20, 43].

High levels of inorganic nitrogen such as ammonia nitrogen and nitrite nitrogen are harmful to fish and are regarded as a limiting factor to production in intensive aquaculture [44]. Compared with residential and industrial sewage, the aquaculture wastewater had its own characteristics with low pollutant concentration and large water flow. Nitrogen, phosphorus concentration, suspended solid content, and the COD of aquaculture wastewater are lower than those of other types of wastewater. Bioflocculant-producing bacteria can use these substances, which are harmful to the growth of fish, and produce bioflocculant with high flocculating activity. These bioflocculant-producing bacteria were successfully used to flocculate particulate and organic matter, improve water transparency and dissolved oxygen, reduce oxygen consumption, and thus improve environment and water quality of aquaculture.

It was of great significance to generate a mutual fusion between the bioflocculant technology of industrial wastewater treatment and biofloc technology of aquaculture to enhance the quality and efficiency of aquaculture and to promote characteristics of Chinese aquaculture that are being friendly to the environment, being healthy, and being sustainable for development.

## 4. Conclusions 

In this study, a bioflocculant-producing bacterium* Bacillus megaterium* SP1 was isolated from biofloc in pond water. The optimal carbon and nitrogen sources for* Bacillus megaterium* SP1 were 20 g L^−1^ of glucose and 0.5 g L^−1^ of beef extract at 30°C and pH 7. Under these optimum culture conditions, the flocculating efficiency of bioflocculant produced by strain SP1 for kaolin suspension was 94.32%. It was demonstrated that adding strain SP1 to aquaculture wastewater could effectively reduce the COD, TAN, and SS and accelerate biofloc formation.

## Figures and Tables

**Table 1:** Screening of bioflocculant-producing bacterium and its flocculating ratio for kaolin suspension.

Number	Flocculating ratio (%)	Number	Flocculating ratio (%)	Number	Flocculating ratio (%)	Number	Flocculating ratio (%)
1	58.2 ± 1.3^e^	12	65.1 ± 3.2^d^	25	68.5 ± 1.9^cd^	*37*	91.9 ± 2.2^a^
2	62.1 ± 1.2^e^	14	54.5 ± 2.1^e^	26	75.9 ± 1.6^c^	38	73.9 ± 1.5^c^
3	66.9 ± 2.2^d^	15	41.9 ± 1.8^g^	27	64.2 ± 2.3^d^	39	78.1 ± 4.2^bc^
4	65.1 ± 0.9^d^	16	51.6 ± 1.1^f^	28	52.5 ± 1.2^f^	40	68.5 ± 3.1^d^
*5*	89.2 ± 0.8^a^	17	66.7 ± 0.8^d^	29	58.1 ± 0.9^e^	41	76.1 ± 2.1^bc^
6	65.1 ± 1.7^d^	18	78.4 ± 2.1^b^	30	62.9 ± 2.1^d^	42	52.1 ± 3.6^f^
7	75.4 ± 1.5^bc^	19	76.1 ± 3.2^bc^	31	57.2 ± 1.7^e^	43	60.3 ± 1.5^e^
8	68.1 ± 1.9^d^	20	70.3 ± 1.5^c^	32	56.6 ± 1.2^e^	44	73.5 ± 0.8^c^
9	62.1 ± 2.1^e^	21	65.2 ± 3.5^d^	33	75.2 ± 4.3^bc^	45	66.5 ± 1.3^d^
10	70.3 ± 0.7^c^	*22*	80.9 ± 2.1^b^	*34*	88.7 ± 3.5^a^	*46*	88.2 ± 2.7^a^
11	42.1 ± 0.6^g^	23	49.9 ± 1.4^f^	35	75.2 ± 2.4^bc^	47	77.9 ± 1.6^b^
12	72.5 ± 1.5^bc^	24	50.2 ± 2.3^f^	36	43.9 ± 1.6^g^	*48*	86.1 ± 2.1^a^

*Note*. Each value represents a mean ± SE (*n* = 3). Values in the line with different superscript letters are significantly different (*P* < 0.05).

**Table 2:** Carbon source metabolic characteristics of SP1 in GN III microplate.

Carbon source reactions	SP1
Polymers	
Dextrin	/
Glycogen	−
Tween 40	///
Sugars and sugar derivatives	
N-Acetyl-d-galactosamine	−
N-Acetyl-d-glucosamine	/
N-Acetyl-*β*-d-mannosamine	−
d-Arabitol	−
d-Cellobiose	−
d-Fructose	/
d-Fucose	/
d-Galactose	/
Gentiobiose	−
*α*-d-Glucose	/
3-Methyl-glucose	−
Myoinositol	−
*α*-d-Lactose	−
d-Salicin	−
d-Maltose	/
d-Mannitol	/
d-Mannose	−
d-Melibiose	−
*β*-Methyl-glucoside	/
Stachyose	−
d-Raffinose	/
l-Rhamnose	−
d-Sorbitol	−
Sucrose	+
d-Trehalose	/
d-Turanose	/
Methyl esters	
Methyl pyruvate	///
d-Lactic acid methyl ester	−
Carboxylic acids	
Acetic acid	−
Acetoacetic acid	/
Citric acid	−
Formic acid	/
l-Galactonic acid lactone	/
d-Galacturonic acid	/
d-Gluconic acid	/
d-Malic acid	−
l-Malic acid	+
d-Glucuronic acid	/
*α*-Hydroxy-butyric acid	−
*β*-Hydroxy-d,l butyric acid	/
*ρ*-Hydroxy-phenylacetic acid	−
d-Saccharic acid	−
Mucic acid	/
Carboxylic acids	
*α*-Keto butyric acid	−
*α*-Keto glutaric acid	−
l-Lactic acid	/
Propionic acid	−
Quinic acid	+
Bromosuccinic acid	/
Amides	
Glucuronamide	//
Amino acids, peptides, related chemicals	
l-Alanine	+
l-Aspartic acid	+
l-Glutamic acid	/
l-Histidine	/
Glycyl-l-proline	−
l-Pyroglutamic acid	/
d-Serine	−
l-Serine	−
d-Aspartic acid	−
l-Arginine	/
*γ*-Amino butyric acid	/
Nucleosides	
Inosine	−
Alcohols	
Glycerol	//
d-Glucose-6-phosphate	−
d-Fructose-6-phosphate	//
Else	
pH 5	+
pH 6	+
1% NaCl	+
4% NaCl	/
8% NaCl	/
1% sodium lactate	+
Fusidic acid	−
Troleandomycin	−
Rifamycin SV	−
Minocycline	−
Lincomycin	−
Guanidine HCl	−
Niaproof 4	−
Vancomycin	−
Tetrazolium violet	/
Tetrazolium blue	−
Nalidixic acid	−
Lithium chloride	/
Potassium tellurite	+
Aztreonam	/
Sodium butyrate	/
Sodium bromate	−

*Note*. +: positive response; −: negative response; /: borderline; //: mismatched positive; and ///: mismatched negative.

**Table 3:** The orthogonal experiment L_16_(4^5^) of optimization of culture conditions.

	A (g L^−1^)	B (g L^−1^)	C (°C)	D	E	Flocculating ratio
1	1 (10.0)	1 (0.2)	1 (20)	1 (7.0)	1	0.785
2	1	2 (0.5)	2 (25)	2 (6.5)	2	0.813
3	1	3 (0.8)	3 (30)	3 (6.0)	3	0.839
4	1	4 (1.0)	4 (35)	4 (5.5)	4	0.765
5	2 (15.0)	1	2	3	4	0.786
6	2	2	1	4	3	0.822
7	2	3	4	1	2	0.806
8	2	4	3	2	1	0.836
9	3 (20.0)	1	3	4	2	0.840
10	3	2	4	3	1	0.866
11	3	3	1	2	4	0.858
12	3	4	2	1	3	0.856
13	4 (25.0)	1	4	2	3	0.788
14	4	2	3	1	4	0.906
15	4	3	2	4	1	0.827
16	4	4	1	3	2	0.808

I	0.800	0.800	0.818	0.838	0.828	
II	0.812	0.851	0.820	0.824	0.817	
III	0.855	0.833	0.855	0.825	0.826	
IV	0.832	0.816	0.806	0.813	0.829	

R	0.055	0.052	0.049	0.025	0.002	

*Note*. A: glucose; B: beef extract; C: culture temperature; D: medium initial pH; and E: blank control.

## Abbreviations

COD: Chemical oxygen demand
TAN: Total ammonia nitrogen
SS: Suspended solids
FV: Volume of biofloc.
